# One Year on: An Overview of Singapore’s Response to COVID-19—What We Did, How We Fared, How We Can Move Forward

**DOI:** 10.3390/ijerph18179125

**Published:** 2021-08-30

**Authors:** S Vivek Anand, Yao Kang Shuy, Poay Sian Sabrina Lee, Eng Sing Lee

**Affiliations:** 1 Ministry of Health Holdings, Singapore 099253, Singapore; VIVEKANA004@e.ntu.edu.sg; 2 Lee Kong Chian School of Medicine, Nanyang Technological University, Singapore 308207, Singapore; SHUY0004@e.ntu.edu.sg; 3 Clinical Research Unit, National Healthcare Group Polyclinics, Singapore 138543, Singapore; Sabrina_Ps_LEE@nhgp.com.sg

**Keywords:** COVID-19, Singapore, pandemic, public health, research

## Abstract

Background—One year has passed since the first COVID-19 case in Singapore. This scoping review commemorates Singaporean researchers that have expanded the knowledge on this novel virus. We aim to provide an overview of healthcare-related articles published in peer-reviewed journals, authored by the Singapore research community about COVID-19 during the first year of the pandemic. Methods—This was reported using the Preferred Reporting Items for Systematic reviews and Meta-Analyses extension for Scoping Reviews (PRISMA-ScR) protocol. It included healthcare-related articles about COVID-19 published between 23 January 2020 and 22 January 2021 with a Singapore-affiliated author. MEDLINE, Embase, Scopus, Web of Science, CINAHL, PsycINFO, Google Scholar, and local journals were searched. The articles were screened independently by two reviewers. Results—The review included 504 articles. Most of the articles narrated the changes to hospital practice (210), while articles on COVID-19 pathology (94) formed most of the non-narrative papers. Publications on public health (61) and the indirect impacts to clinical outcomes (45) were other major themes explored by the research community. The remaining articles detailed the psychological impact of the pandemic (35), adaptations of medical education (30), and narratives of events (14). Conclusion—Amidst a resurgence of community cases involving variant COVID-19 strains, the resources from the research community will provide valuable guidance to navigate these uncertain times.

## 1. Introduction

The COVID-19 virus first arrived in Singapore on 23 January 2020 via an imported case of infection [1]. Since then, the pandemic progressed to a peak of large clusters involving dormitories which warranted the implementation of a two-month-long circuit breaker. (The circuit breaker was a stopgap measure enacted by the Government involving movement restriction of members of the public for non-essential reasons during the initial phases of the pandemic. These measures were implemented between 7 April 2020 and 2 June 2020 and were succeeded by a multi-stage re-opening termed Phase 1 to 3.) As cases began to fall, a cautious and calculated series of re-opening measures across a span of three phases were implemented to allow for a controlled return to day-to-day living. These re-openings were supported by various milestone events such as the completion of worker dormitory testing [2], and a renewed confidence reflected in the selective opening of borders to other countries [3]. Singapore’s vaccination campaign started near the end of 2020 [4] and has since gained significant traction, with about a third of the population being fully vaccinated [5]. Inevitably, there were challenges along the way, with a sudden spike in community cases and local detection of the more infectious Delta strain [6] necessitating a state of heightened alert. (A heightened alert is a state of increased restrictions on social gatherings and workplace measures implemented due to worsening trends in viral transmission. These reflect a temporary reversal of earlier re-opening measures while still allowing limited activities to continue.) Since then, with the collective contributions of the Singapore population, the country has been back on track to wrestle the pandemic under control.

The first year of the pandemic saw Singapore face significant uncertainty treading through uncharted waters. The various Association of Southeast Asian Nations (ASEAN) countries in the region also faced similar situations and were struggling with high rates of COVID-19 spread with not much known about this new threat. There were significant obstacles faced by the Singapore research community. Many clinical and experimental research projects, especially those requiring face-to-face interaction or requiring the collection of data across various institutions had to be cancelled or postponed. Even as the nation re-opened, additional policies, such as split-team working arrangements, made continuing with research a challenging affair. In spite of this period of uncertainty, the research community stepped forward to produce quality literature on various aspects of the pandemic, with findings that were relevant to healthcare institutions, the country, and the global community, both in the present and for the future. Despite the ever-evolving crisis creating new questions to be answered at every turn, the research community rapidly adapted and produced articles that made great headways in tackling the pandemic. Singapore has also recently advanced a COVID-19 research collaboration with the regional ASEAN countries to help forge ahead with quality research in this pandemic [7]. This will no doubt go a long way in helping to bring the pandemic under control within the region, given the current shared challenges faced by the more transmissible Delta variant.

This paper aims to provide an overview of healthcare-related articles published in peer-reviewed journals that were authored by the Singapore research community about COVID-19 during the first year of the pandemic. By doing so, we hope to identify areas which have shown significant progress in terms of articles published, as well as areas where more research is needed.

## 2. Materials and Methods

This scoping review was reported using the Preferred Reporting Items for Systematic reviews and Meta-Analyses extension for Scoping Reviews (PRISMA-ScR) protocol [8].

Articles were included if they were (a) healthcare-related articles with content relating to COVID-19, (b) published from 23 January 2020 to 22 January 2021, (c) conducted in any country of origin but has at least one Singapore-affiliated author who is either the first, second, or last author, with Singapore being the primary affiliation if the author has multiple affiliations, and (d) were available on online databases. Articles were excluded if (a) they were not published in peer-reviewed journals, (b) published in any language other than English, (c) unrelated to healthcare or medical research, or (d) if they were conference proceedings, protocol papers, or preprints.

The bibliographic databases of MEDLINE, Embase, Scopus, Web of Science, CINAHL, PsycINFO, and Google Scholar were searched for all the records from 23 January 2020 to 22 January 2021 to identify potentially relevant articles. The search strategies were drafted and refined through a team discussion amongst the authors and the librarian from the Lee Kong Chian School of Medicine. The final search strategy for the seven databases can be found in Supplementary Table S1. Additionally, local journals, such as the *Annals of the Academy of Medicine Singapore* and the *Singapore Medical Journal,* were manually searched for relevant articles over the above stated time period. The final search results were exported into EndNote where duplicate records were removed. These searches were supplemented by hand searches of the references listed in the included articles. This was an iterative process, repeated until no new articles were identified.

The articles were screened by two independent reviewers (S.V.A. and Y.K.S.) using Covidence, who then extracted the data from the selected articles into a spreadsheet and categorised them. The reviewers sequentially evaluated the titles, abstracts, and then full texts of all the publications identified by the searches for potentially relevant articles. The spreadsheet captured the relevant information on publication details (e.g., first author, publication date), conduct of the study (study design, institution), and article content (the main research question and answer, as well as important discussion points). The spreadsheet also included reasons for the exclusion of articles. The articles were categorised based on the content of the article into one of the following topics: (a) changes in hospital practice, (b) COVID-19 pathology, (c) impact of the COVID-19 pandemic on clinical outcomes, (d) public health, (e) psychological impact, (f) medical education, (g) narrative of events, or (h) others. They were further sub-categorised based on recurring themes within each category. The papers were categorised based on their content and main summary points. Two of the study authors read each of the papers in full. As the data was extracted from the papers, categories were being generated. Similar articles would be grouped together, while new categories would be generated if an article did not fall into existing categories. When there were conflicts of assigned categories, they were resolved after a discussion with all four study authors. The categories were later merged to form larger, more noteworthy groups. In this review, we include research derived from sources ranging from hospitals, academic institutions, and government bodies to increase coverage of articles produced by the Singapore research community.

## 3. Results

**Figure 1:**
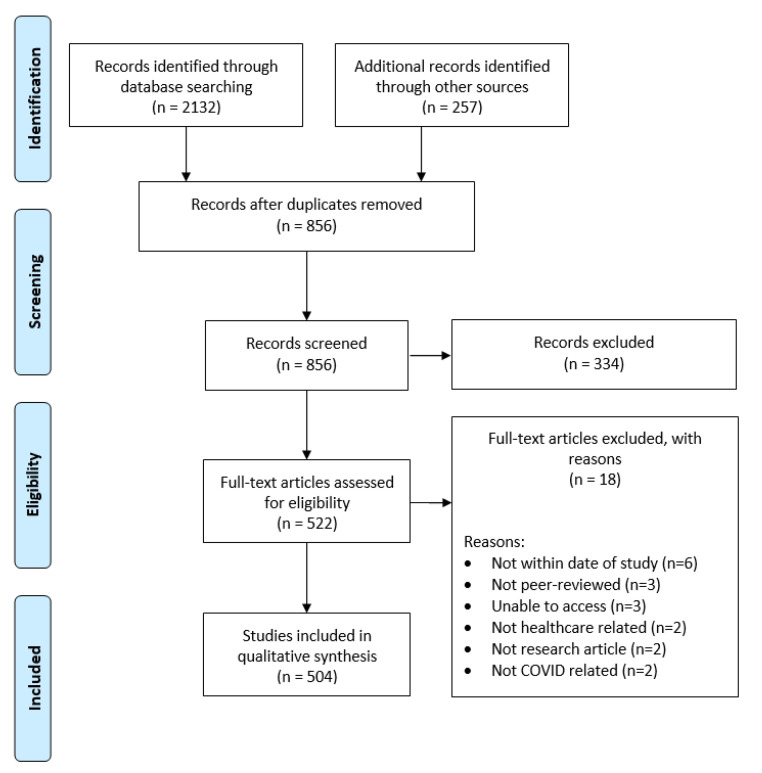
Preferred Reporting Items for Systematic reviews and Meta-Analyses (PRISMA) flow diagram.

A total of 2389 records were obtained from the searches as well as other sources before duplicate records were removed. A total of 522 full text articles were assessed for eligibility (Figure 1). Of these, 18 were excluded for reasons shown in Figure 1. The remaining 504 articles were selected for this scoping review.

**Figure 2:**
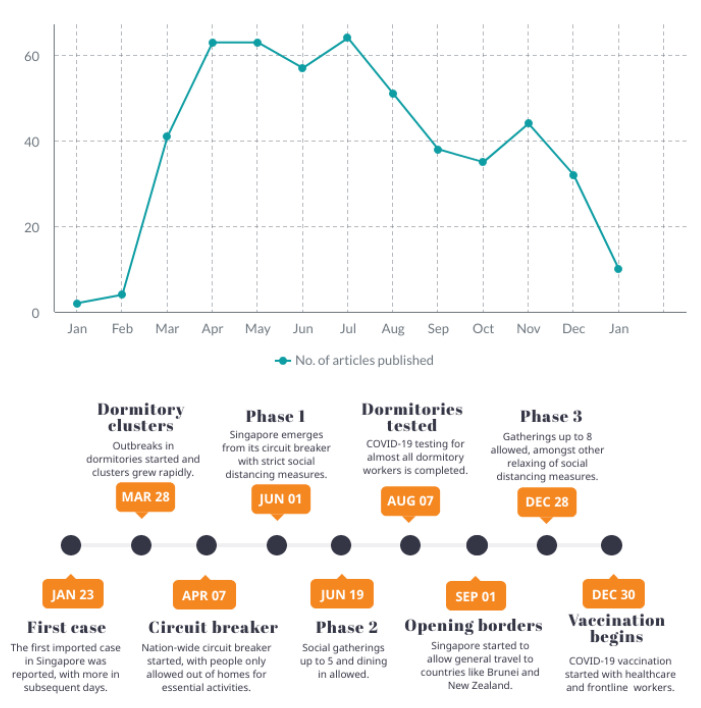
Articles published per month and a timeline of the first year of the COVID-19 pandemic.

Figure 2 shows the number of articles published per month over the time range of this scoping review (23 January 2020 to 22 January 2021), with a corresponding timeline of major events in Singapore. The major events were chosen due to their impact on residents’ lifestyle in Singapore and the corresponding changes in the healthcare practices. These milestones reflect the changing state of the pandemic in Singapore over the last year, which were likely to have dynamic implications on the public perception of the threat of COVID-19.

The first spike of publications occurred three months after the pandemic arrived on Singapore’s shores, with significant interest on the subject being maintained for four months. This was succeeded by a steady decline in publications over the subsequent months. It is hypothesised that the later decline in articles may be an effect of a reduction in the “changes in hospital practice” articles, which inflated the initial publication numbers. The focus and priorities of the local research is likely to have evolved around the changing demands of the pandemic, with a diversion of resources to other topics of interest.

**Figure 3:**
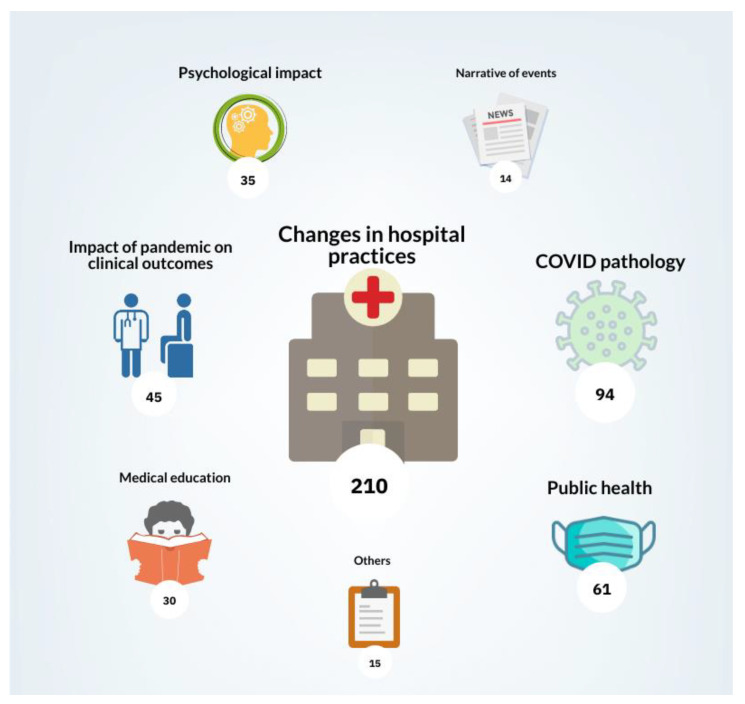
Articles published per month and a timeline of the first year of the COVID-19 pandemic.

The 504 articles were categorised into eight themes based on their content, as shown in Figure 3. The categories were identified as they were prominent recurring themes among the articles, suggestive of topics that were of great pertinence to the pandemic. Articles describing the changes in hospital practices were the most common, followed by themes of COVID-19 pathology and public health.

The 504 articles were further sub-categorised, as presented in Table 1. The sub-categories were created by similarly identifying the recurring sub-themes within each category.

### 3.1. Changes in Hospital Practice (210 Articles)

The biggest contributors in this category were from surgical specialties [9,10,11,12,13,14,15,16,17,18,19,20,21,22,23,24,25,26,27,28,29,30,31,32,33,34,35,36,37,38,39,40,41,42,43,44,45,46,47,48,49,50,51,52,53,54,55,56,57,58,59,60,61,62,63,64,65,66,67,68,69,70,71,72,73,74,75,76], with general surgery [17,18,19,20,21,22,23,24,25,26,27,28,29,30,31,32] being the most active in producing articles. The internal medicine departments [77,78,79,80,81,82,83,84,85,86,87,88,89,90,91,92,93,94,95,96,97,98,99,100,101,102,103,104,105,106,107,108,109,110,111,112,113,114,115,116,117,118,119,120] were led by cardiology [86,87,88,89,90,91,92,93] and oncology [113,114,115,116,117,118,119,120]. There were some contributions by allied health and dentistry departments [121,122,123,124], with an article on nursing [123] and occupational therapy [124]. Out of the remaining departments [125,126,127,128,129,130,131,132,133,134,135,136,137,138,139,140,141,142,143,144,145,146,147,148,149,150,151,152,153,154,155,156,157,158,159,160,161,162,163,164,165,166,167,168,169,170,171,172,173,174,175,176,177,178,179,180,181,182,183,184,185,186,187,188,189,190], the majority of the articles were from the radiology department [175,176,177,178,179,180,181,182,183,184,185,186,187,188,189,190]. It is worth noting that dedicated articles on the adoption of telemedicine [191,192,193,194,195,196,197,198,199,200,201,202,203,204] were also published, with populations of child and adolescent telemedicine [195,197] and that of older adults [194,196,204] being discussed separately.

These articles were narratives describing the adaptations made by various hospital departments in Singapore to allow for the delivery of timely and safe healthcare during the COVID-19 pandemic (number of articles listed in brackets). The most frequently mentioned topic was personal protective equipment (PPE) or powered air-purifying respiratory (PAPR) procurement, rationing, and usage across practice (154). However, significantly fewer articles actively reinforced the importance of PPE/PAPR refresher training (59), and the importance of hand hygiene practices for healthcare workers (HCWs) (38). Another common sub-category described the implementation of isolation and safe distancing measures in healthcare settings (119). Another common topic was changes to deployment of staff, where both intra-departmental and inter-departmental modifications were noted (125). Of these changes, the majority explicitly highlighted intra-departmental team segregation that aimed to ensure business continuity in the event of COVID-19 transmission among the staff (91). The screening of the health conditions of patients (82) and staff (59), alongside changes in visitation policies (53) were also implemented by various departments.

Specifically with regards to the changes in clinical practice, several articles reported a need to reduce the listings of elective surgeries or delay non-essential outpatient visits as a means of increasing the hospital spare capacity (107). The use of telemedicine services to divert the clinic workload and improve care was also explored (79), along with the introduction of home medicine delivery services (7). Video conferencing platforms were also used to replace physical meetings for clinical and educational purposes (58). The pandemic has also resulted in changes to the clinical management of patients (48), such as with early intubation [91]. Changes were also made to the operating theatre (OT) setup, with the most commonly cited adaptation being the use of negative pressure rooms for COVID-19 cases [30,134]. Discussions on OT air changes per hour [147,148] and adoption of high-efficiency particulate air (HEPA) filters [175] were commonly cited as well. Managing patient transfer within and between hospitals was also an important consideration (35). Simulations and training sessions were important in identifying gaps in the existing workflows (18), serving to refine the aforementioned changes made.

The measures that have an indirect impact on clinical care were also brought up. Improving communication channels between the hospital leadership and HCWs (53) allowed workers on the ground to respond quickly to the changing conditions of the pandemic, while initiatives to maintain the HCWs’ morale was important to minimise burnout (38). These measures do not immediately influence the pandemic response but can generate a compounding impact on the resilience of frontline workers during the pandemic, with the spillover effects on other prongs of the pandemic response plan.

### 3.2. COVID-19 Pathology (94 Articles)

The most common type of articles in this category were case reports and those about the symptoms, signs, and complications of COVID-19 [204,205,206,207,208,209,210,211,212,213,214,215,216,217,218,219,220,221,222,223,224,225,226,227,228,229,230,231,232,233,234,235,236,237,238,239,240,241,242]. These articles described the various ways in which COVID-19 could present, such as olfactory loss [206,209,214,219], radiological findings [217,221,231,232], and various other presentations. Viral transmission of COVID-19 was the next most commonly studied [93,243,244,245,246,247,248,249,250,251,252,253,254,255], which looked at the various modes of transmission [245,247,249,252,253,254], and contamination of the environment and PPE [243,244,248]. COVID-19 diagnostic testing [256,257,258,259,260,261,262,263,264,265] was also discussed, from the current testing processes available [256,257,258,259,261,264,265] to the workflows for diagnosis [260,262,263]. Some articles described COVID-19 treatment [266,267,268,269,270,271,272,273,274,275,276], ranging from assessing the pharmacological options [274,276] to other forms of management, such as prone positioning [267,270]. Various aspects of the molecular pathology of COVID-19 [277,278,279,280,281,282,283] were studied as well, such as the T-cell response [277] and the viral spike protein [278]. There were a few articles about general COVID-19 pathology [284,285,286,287], which included combinations of topics from the above sub-categories. Another few were about co-infection with other pathogens, such as the human immunodeficiency virus (HIV) [288,289] and mycoplasma pneumoniae [290]. The remaining articles were about the various other aspects of COVID-19 pathology not mentioned above [291,292,293,294,295,296], such as dealing with a dual outbreak of dengue and COVID-19 [292], and respiratory surveillance wards to detect COVID-19 early [296].

### 3.3. Public Health (61 Articles)

Healthcare worker safety was the most common topic in this category [297,298,299,300,301,302,303,304,305,306,307,308,309]. These articles described the use of PPE amongst HCWs for protection [297,301,302,307,308], the adverse effects from prolonged PPE use and hand hygiene [299,305,306], as well as the measures to prevent transmission amongst HCWs [303,304,309]. Several articles were about the containment of COVID-19 within the general population [310,311,312,313,314,315,316,317,318,319,320], such as the steps following the circuit breaker [312,314], as well as an evaluation of Singapore’s containment efforts, from surveillance and isolation [310,315,316,317,318] to managing the outbreak at dormitories [319,320]. Some articles described the transmission of COVID-19 in the population [321,322,323,324,325,326,327,328], including household [321,324] and asymptomatic transmissions [326,327]. There were articles about the epidemiological investigations into the clusters of COVID-19 cases [329,330,331,332,333], as well as some about the evaluation of methods of contact tracing, including the TraceTogether mobile application [334,335,336,337,338,339]. The effectiveness of the screening and testing strategies employed for COVID-19 were also discussed [340,341,342,343,344]. A few articles were about compliance to the public health measures [345,346,347], and statistical modelling [348,349,350] to estimate the reproductive number and rate of spread of COVID-19. The remaining articles were about other aspects of public health, such as how community care facilities [351] and the National Centre for Infectious Diseases (NCID) [352] were initiated.

### 3.4. Impact of Pandemic on Clinical Outcomes (45 Articles)

The majority of the articles in this category were about the impact of COVID-19 on conditions relating to specialties within internal medicine [353,354,355,356,357,358,359,360,361,362,363,364,365,366,367,368,369,370,371,372,373,374,375,376,377,378,379]. They explored areas such as the reduction in respiratory infections [365,368,369,370,371,374,375,379,380], stroke admissions [376,377], increase in the door-to-balloon time for percutaneous coronary intervention [358], and the impact on the care of patients with dementia [362,364]. Several articles were about surgical specialties [381,382,383,384,385,386,387], such as the management of cholecystitis [382], the outcomes in pregnant women infected with COVID-19 [384], and the decrease in hip fractures [385]. The impact on conditions related to other specialties [388,389,390,391,392,393] was also described, such as the reduction in the number of patients seen at the emergency department [394], the epidemiological trends in Kawasaki disease [389], and the management of patients with chronic pain [395].

### 3.5. Psychological Impact (35 Articles)

The most common topic was the psychological impact of COVID-19 on HCWs [396,397,398,399,400,401,402,403,404,405,406,407,408,409,410,411,412,413] (18), which explored burnout at work [396,401,405] and methods to address this psychological impact [404,409]. The impact on patients [414,415,416,417,418,419,420] was also explored (7), looking at pregnant women [415,416] and cancer patients [420]. The remaining articles looked at the impact on the general public [421,422,423,424,425,426,427,428,429,430], such as their psychological wellness during this pandemic [421,422,423,425,428,430], the adaptation of school community mental health services [429], and learning from the psychological responses and coping methods in previous outbreaks [427].

### 3.6. Narrative of Events (14 Articles)

The majority of the articles were a summary of events [431,432,433,434,435,436,437], describing the key events and measures taken on a societal level in response to the pandemic. Comparisons with previous pandemics and outbreaks such as those of the severe acute respiratory syndrome (SARS) and Nipah viruses were analysed [438,439,440,441,442], to highlight the learning points that could be applied to the COVID-19 pandemic. Comparisons with the responses of other countries were also made [443,444], emphasising the lessons that could be learnt from them. 

### 3.7. Medical Education (30 Articles)

About half the articles were related to undergraduate medical education [445,446,447,448,449,450,451,452,453,454,455,456,457,458], such as the switch to online learning [446,447,448,449,450,452,453,455,456] and resuming clinical placements [454,458] in an attempt to minimise the disruption to clinical learning. The remaining half were about residency training [459,460,461,462,463,464,465,466,467,468,469,470,471,472,473,474], which explored the impact of the pandemic on various specialties’ training programmes [462,463,464,465,466,468,469,472], and the well-being of residents [460,467,474] during these trying times.

### 3.8. Others (15 Articles)

Several articles were about the roles or impact on specific populations during the pandemic [475,476,477,478,479,480]. These included migrant workers [477,478,479,481], the general public [476], surgeons [480], and dentists [475]. Research activities during the pandemic were discussed [482,483,484,485], looking at the current gaps in knowledge and how to further progress the research about COVID-19 [483,484], as well as the impact of the pandemic on the conduct of research [482,485]. Some articles were about medical ethics [486,487,488,489], exploring ethics in general [487], inequities in healthcare among the population [489], and the allocation of resources for critical care in this pandemic [486,488].

## 4. Discussion

Overall, the Singapore research community has published 504 healthcare-related articles about COVID-19 during the first year of the pandemic. The initial peak of articles coincided with the implementation of the circuit breaker measures, suggesting that COVID-19-related research in Singapore gained traction as the intensity of the pandemic rose. The interest in such publications was maintained over the next few months, during which the COVID-19 containment measures were at their most stringent. It can be seen that the Singapore research community has been highly adaptive to the state of the pandemic, progressing the collective knowledge base where it is of the greatest necessity.

### 4.1. Changes to Hospital Practice

The majority of the articles were about changes in hospital practice, which could have served as a useful reference for other countries looking to implement measures to minimise the spread of COVID-19 while continuing clinical practice. The steps taken in local hospitals were largely similar to those in other countries [490,491,492,493,494], which emphasised the widespread acceptance of the necessity of such measures. It is likely that these measures contributed to minimising the spread of COVID-19 in Singapore, with early implementation and an efficient execution. These articles collectively reinforce that these common measures enacted can be rapidly integrated into different healthcare systems with similar results. It is, however, important to note that the nature of the pandemic differs from country to country. Singapore was fortunate to have no hospital COVID-19 clusters during the first year of the pandemic, up till 27 April 2021 [495], while hospital clusters were prevalent in Taiwan [496], the United States [497], and the United Kingdom [498].

Telemedicine gained a sharp rise in interest during the pandemic as a result of clinics postponing the non-urgent caseloads to increase the capacity to respond to the requirements of the pandemic. Major leaps were made as telemedicine was explored by many departments in the triage, diagnosis, and clinical management of less complex conditions. Telemedicine possesses immense potential to remain a mainstay in healthcare in a post-pandemic setting. The fields that usually emphasised significant in-person contact, ranging from physiotherapy to psychiatry, have found success in conducting treatment programmes on remote platforms with comparable results to the in-person services [499,500]. Telemedicine is proving to be an effective complement to traditional face-to-face clinics and is expected to make significant headway in the future. Studies have demonstrated that post-stroke telerehabilitation produced comparable results in achieving patient outcomes compared to traditional telemedicine [501]. Further studies should monitor the impact of telemedicine on long-term patient morbidity and patient satisfaction in Singapore. This produces metrics to evaluate this up-and-coming addition to the healthcare service.

There is room for more research by allied health practitioners on how the pandemic has influenced their workflow, so that others may learn from their experiences to improve hospital protocols.

### 4.2. COVID-19 Pathology and Diagnosis

The large number of articles on COVID-19 pathology also helped to shed light on the nature of this novel virus early on in the pandemic, when much was unknown about it. The evidence put forward in these articles, such as the signs and symptoms of COVID-19, modes of transmission, diagnostic testing, and efficacy of treatment options, among others, complemented the literature produced by the countries that were first exposed to the virus. The fact that such a comprehensive body of knowledge about COVD-19 was built up in this short span of time is a sign of the tireless efforts of the research community over the last year. These findings went a long way in helping inform the national and hospital guidelines to contain the spread of the virus, as well as to give COVID-19 patients the best standard of care backed by evidence.

Clinicians are now aware of the diversity of presentations of COVID-19 ranging from anosmia to thromboembolic events. Regarding the diagnosis of COVID-19, the studies have shown that the existing testing modalities, including polymerase chain reaction (PCR) and immunoassays, have good diagnostic value, and can continue to be used while better methods are being developed. Nasopharyngeal specimens, followed by throat specimens, offer the highest clinical sensitivity for COVID-19 diagnosis in early illness. The clinical sensitivity improves and is similar when either mid-turbinate or nasopharyngeal specimens are combined with throat specimens [258]. The serological assays available currently target a diverse range of the viral antigen which can be used to assist in the accurate diagnosis of COVID-19 [265]. Regarding the transmission of the virus, given that there is evidence for transmission via environmental contamination, care should be taken to disinfect environments of confirmed cases. The transmission through air also supports the use of the current mask wearing policies as a minimum amongst the public [244]. The incubation period of locally acquired COVID-19 cases ranged from 1 to 12 days with a median of 5 days, supporting the use of 14 days as a quarantine period for suspected cases [250]. Regarding the treatment of COVID-19, it is still a fresh area of evolving research, but early studies have shown prone positioning to be a low-cost and effective measure in improving outcomes [267,270].

With the advancement of new technology, the efficacy of newer diagnostic and therapeutic modalities are yet to be described and reported at the point of this paper. Furthermore, new strains that are of global concern, such as the Delta variant, would need to be further investigated. Improved details of its transmission characteristics as well as how effective the existing treatment regimens are against it could help guide policy making to deal with this highly infectious strain.

### 4.3. Public Health

The articles on public health also offered a detailed analysis on the current measures taken, most notably the contact tracing and containment measures, which identified the areas that were working well, as well as those which required further refinement. For instance, the use of digital contact tracing via TraceTogether was noted to originally have low take-up rates by the elderly, which was later addressed by creating tokens for them [337] as well as other groups of individuals without constant access to smartphones, such as young children and students [502]. In another example, a case report detailed how an imported case passed through our borders, and highlighted the gaps in public health and border control strategies which have since been effectively addressed, such as raising awareness of the less common symptoms of COVID-19 to declare, and transporting such visitors separately to dedicated residential areas to serve their quarantine [311]. Such articles will be especially valuable in the current heightened alert (May–June 2021) situation where the Delta variant has become the most prevalent strain among local community cases [6]. With the variant being estimated to be 60% more contagious than other strains [503], the implications of public health research will be compounded and be of increasing relevance as the pandemic evolves alongside a dynamic environment. The research conducted over the past year will be of great aid in dealing with the resurgence of the virus now and in the future. The articles detailing how contact tracing and community care facilities have successfully helped stem the spread of the virus locally [310,315,319,334,335,336,337,338,339,351] can be used as a reference for other countries to complement the existing public health measures and gain better control of the pandemic situation.

The tracking and identifying of clusters is important in allowing us to identify the transmission dynamics of the virus, allowing us to devise suitable public health measures to minimise local community transmission, such as reinforcing the importance of social distancing and isolation of COVID-19 positive patients [329] and being better equipped to identify the COVID-19 cases that might be missed by other surveillance methods [330]. There is also a need to rely on but not be over-dependent on digital contact tracing, but it cannot be used to completely replace the other aspects of the nationwide COVID-19 screening strategy [334,338]. Given the scale of the pandemic, a multi-pronged containment strategy that includes stay home notices (SHN) [317], isolation facilities [319], telemedicine [319], and self-monitoring [319] is required to effectively and efficiently manage the spread of the pandemic.

An emerging area of importance is how to improve the vaccination rates now that the vaccination supply is better. As vaccinations prove to be a vital aspect in controlling the spread of the pandemic, studies should investigate the concerns raised by members of the public that reject vaccinations, as well as explore effective solutions to encourage vaccinations. The existing contact tracing measures/containment measures also need to be evaluated for their effectiveness in the longer term. While they may have served us well in the first year, the emergence of new clusters and rising case numbers in recent times indicate that our public health measures are not foolproof.

### 4.4. Impact of COVID-19 Pandemic on Clinical Outcomes

It is apparent through these articles that this pandemic also has far reaching indirect effects on clinical outcomes. While some effects such as the reduction in respiratory viral illnesses [365,368,369,370,371,374,375,379,380] are a welcome change, others such as the increase in the door-to-balloon time for percutaneous coronary intervention [358], the impact on cardiac rehabilitation services [354] and community services for patients with dementia [364], as well as a reduction in childhood vaccination rates [390] are a cause for concern. These highlight the importance of identifying such vulnerable groups of patients affected by the pandemic, and the need to take targeted measures to address these issues. These articles remind us that every measure enforced during the pandemic will have unforeseen indirect consequences which must be properly managed to avoid impeding the delivery of quality healthcare. Failing to act on this now will result in far reaching consequences in the future, with increased morbidity and mortality for these groups of patients.

While these articles mostly described the limitations in care provided to patients as a result of the COVID-19 infection control measures, future studies should investigate the impact of these changes on patient recovery and mortality. In particular, the switch of various services to telemedicine modalities needs to be carefully considered and evaluated based on the patient care outcomes.

### 4.5. Psychological Impact

The psychological impact of the pandemic was not neglected by the research community. There is clear and significant evidence that COVID-19 has led to increased HCW stress, burnout, perception of stigma [396,404,405], and that interventions to improve the mental health of HCWs are beneficial [397,399,407,408,412]. COVID-19 lockdown and isolation measures have also led to poorer mental health of the general population, hence, psychological interventions should be considered a fundamental aspect of any pandemic plan [421,426,427,428,430].

A substantial number of the articles explored in detail how HCWs, patients, and the general community faced challenges such as burnout in particular, as well as explored suitable coping measures. This is consistent with the articles published overseas, which also found significant burnout amongst healthcare workers [504,505,506,507] and adverse psychological effects on the rest of the community [508,509,510,511]. The articles published locally have detailed various responses to this situation, such as a digital MyCare application [417], a psychological preparedness kit for HCWs [409], and peer support services [430], which demonstrate that our community has been able to anticipate and react quickly to the psychological impact of this pandemic. Given that this pandemic is set to last for some time, dealing with the psychological sequela is equally as important as managing the physical effects of an infection. While this impact of COVID-19 has since been well established and gaining interest, more studies should be conducted to investigate the efficacy of the interventions used to mitigate the psychological impact of COVID-19, as failure to manage this adequately will lead to poor outcomes for mental health.

The long-term impact of COVID-19 survivors can be looked into. There are many studies looking into the impact of COVID-19 on the mental health of HCWs and the general public. However, future efforts should be focused on devising effective solutions to tackle the decline in mental health as a result of the pandemic to cope with this increasingly known sequela of the pandemic.

### 4.6. Medical Education

Looking ahead, sustaining medical education during the extended pandemic is of great interest in this pandemic as well. A careful balance needs to be struck between reducing the exposure of medical trainees to unnecessary risks in the hospital, and concurrently being mindful in minimising the impact on their training. Interruptions in training will delay the pipeline sustaining the healthcare system, leading to snowballing impacts on manpower and HCWs’ competencies. While bedside clinical teaching hours will inevitably decline during the pandemic, there is abundant potential to maximise the hours freed up for other productive learning opportunities. Our educational institutions should continue to meaningfully engage medical students and residents during this pandemic, as valuable lessons can still be learnt from adapting to this ever-evolving situation, ensuring that there is no compromise to the standard of care provided by healthcare institutions.

Additional studies can focus on batches of medical students whose clinical postings were affected by this pandemic, to evaluate if their clinical skills and knowledge have managed to catch up with the additional measures put in by medical schools to mitigate the impact of this pandemic.

### 4.7. Comparison with Similar Studies

Studies similar to our scoping review were conducted and were described in the earlier section [431,432,433,434,435,436,437]. These works highlighted the earlier phases of Singapore’s response towards the pandemic but did not cover the same breadth of categories that were discussed in this study. These papers most commonly discussed the aspects of public health measures to curb the spread of COVID-19, with limited focus on the experimental and clinical research outside of descriptive studies. The findings from these papers have been summarised in Section 3.7.

### 4.8. Limitations

We did not critically appraise the quality of each study as we expected a heterogenous variety of studies. Moreover, a large proportion of the studies were narrative descriptions of the changes made in hospitals and society at large. However, these were sufficiently detailed to allow other hospitals/countries to replicate the changes or implement the key aspects of them in guiding policy making, hence, there is minimal variation in the robustness of these studies. There were several observational studies looking at the impact of the pandemic on clinical outcomes. The sample size for these studies was usually restricted to patients from a given hospital over a few months. Given that the pandemic had only been going on for about a year at the time of this scoping review, these studies offered useful knowledge based on the limited information available at that time. Finally, there were several case reports about the presentation of COVID-19—which by themselves were limited, but when taken together as a whole and considered alongside the evidence reported in other countries, can add value to our knowledge base about COVID-19.

## 5. Conclusions

In conclusion, the Singapore research community has published numerous healthcare-related articles over the last year, which will go a long way in helping to deal with the various facets of this pandemic. The changes in hospital practice, the impact on clinical outcomes, COVID-19 pathology, medical education, and the psychological impact of the pandemic were some areas that were thoroughly investigated. While providing an overview of the published articles over the last year, this paper has also highlighted some areas where more research will be needed in the future. Studies would need to be conducted to assess how effective the current measures adopted in hospitals have been in preventing the spread of COVID-19. More research into the transmission and management of the Delta variant and other new variants would also be necessary. On the public health front, another area that would need further studies is how to improve COVID-19 vaccination rates, as well as an analysis of the current contact tracing methods in light of more transmissible variants. More research would also need to be conducted to assess the longer-term impact of the pandemic-related changes in clinical practice on patient outcomes, including the efficacy of telemedicine. In terms of the psychological impact of the pandemic, studies on the mental well-being of COVID-19 survivors is one area not fully explored yet. The impact of the pandemic on medical education in the longer term would also need to be studied, to assess how the knowledge and skills of the next generation of clinicians might be affected.

This scoping review can be a used as a precursor to a systematic review. The future direction of such a review would focus on the areas of emerging importance in the pandemic. These include vaccination (evidence on the various types, efficacy against the various strains, and likely duration of effect) and public health measures (sustainable measures as we move into an era of living with the pandemic), as well as faster and simpler COVID-19 screening methods.

As the pandemic continues, the important areas of focus will also evolve and change. We have put forward the body of work contributed by Singapore’s research community over a one-year period to the international community in keeping up with the dynamic changes caused by COVID-19 globally. The findings from this review are a salutation to the efforts of the research community to steer this pandemic to an eventual close. When that day arrives, the work of researchers during the pandemic will be looked back on with immense gratitude for its vast contribution towards making this outcome possible.

## Figures and Tables

**Table 1:** An overview of the healthcare-related peer-reviewed articles published by the Singapore research community about COVID-19 during the first year of the pandemic.

Theme	No. of Articles	Theme	No. of Articles	Theme	No. of Articles
**Changes in hospital practice**	**210**	**COVID-19 Pathology**	**94**	**Public Health**	**61**
General changes to practice	14	Case reports, symptoms and complications	39	Healthcare worker safety	13
Telemedicine	13	Viral transmission modalities	14	Containment	11
Internal Medicine	46	Testing	10	Transmission across population	8
Surgical	69	Treatment	11	Contact Tracing	6
Other specialties	64	Molecular pathology	7	Clusters	5
Allied Health/Dentistry	4	General	4	Screening and Testing	5
		Co-infection	3	Compliance	3
**Impact of pandemic on clinical outcomes**	**45**	Others	6	Modelling	3
Internal Medicine	28			Others	7
Surgical	7	**Narrative of events**	**14**		
Other specialties	10	Summary of events	7	**Others**	**15**
		Comparison with previous pandemics	5	Study of a population	7
**Psychological impact**	**35**	Comparison with other countries	2	Impact on research	4
Healthcare workers	18			Ethics	4
Patients	7	**Medical education**	**30**		
General population	10	Undergraduate	14		
		Residency training	16		

The eight main themes of the articles are highlighted in bold.

## Data Availability

Not applicable.

## Supplementary Materials

The following are available online at https://www.mdpi.com/article/10.3390/ijerph18179125/s1, Table S1: OVID MEDLINE; Table S2: Embase; Table S3: Scopus; Table S4: Web of Science; Table S5: CINAHL; Table S6: Psycinfo; Table S7: Google Scholar.
