# Supplementary material for: One Year on: An Overview of Singapore’s Response to COVID-19—What We Did, How We Fared, How We Can Move Forward
Source: Int J Environ Res Public Health. 2021 Aug 30;18(17):9125. doi: 10.3390/ijerph18179125 (PMC8431401; doi:10.3390/ijerph18179125)
Supplement: Supplementary file 1 [file ijerph-18-09125-s001.zip › ijerph-1306940-supplementary.pdf]

| Supplementary Table S1: OVID MEDLINE | | |
|---|---|---|
| # | Searches | Results |
| 1 | exp Coronavirus Infections/ or exp Pneumonia, viral/ or exp Pandemics/ | 65837 |
| 2 | (COVID-19 OR COVID19 OR 2019 Novel Coronavirus OR 2019-nCoV OR nCoV-19 OR Coronavirus Disease 2019 OR Coronavirus Disease-19 OR SARS Coronavirus 2 OR SARS-CoV-2 OR Severe Acute Respiratory Syndrome Coronavirus 2).mp | 99632 |
| 3 | Singapore/ | 13644 |
| 4 | Singapore.kw,ti | 8146 |
| 5 | 1 or 2 | 120182 |
| 6 | 3 or 4 | 15279 |
| 7 | 5 and 6 | 610 |
| 8 | Limit 7 to yr="2020-2021" | 374 |

| Supplementary Table S2: Embase | | |
|---|---|---|
| # | Searches | Results |
| 1 | exp pandemic/ or exp Coronavirinae/ | 67089 |
| 2 | (COVID-19 OR COVID19 OR 2019 Novel Coronavirus OR 2019-nCoV OR nCoV-19 OR Coronavirus Disease 2019 OR Coronavirus Disease-19 OR SARS Coronavirus 2 OR SARS-CoV-2 OR Severe Acute Respiratory Syndrome Coronavirus 2).mp | 96937 |

| | | |
|---|---|---|
| 3 | Singapore/ | 23819 |
| 4 | Singapore.kw,ti | 10683 |
| 5 | 1 or 2 | 120861 |
| 6 | 3 or 4 | 25378 |
| 7 | 5 and 6 | 645 |
| 8 | Limit 7 to yr="2020-2021" | 483 |

| **Supplementary Table S3: Scopus** | | |
|---|---|---|
| # | Searches | Results |
| 1 | TITLE-ABS-KEY ( Covid-19 OR Covid19 OR (2019 Novel AND Coronavirus ) OR nCov-19 OR ( Coronavirus AND Disease 2019 ) OR ( Coronavirus AND Disease-19) OR ( SARS AND coronavirus 2 ) OR SARS-COV-2 OR ( Severe AND Acute AND Respiratory AND Syndrome AND Coronavirus 2 ) ) | 108950 |
| 2 | TITLE ( Singapore ) OR KEY ( Singapore) | 36481 |
| 3 | 1 AND 2 | 514 |
| 4 | 3 AND ( LIMIT-TO ( PUBYEAR , 2021 ) OR LIMIT-TO ( PUBYEAR , 2020 ) ) | 504 |

| **Supplementary Table S4: Web of Science** | | |
|---|---|---|
| # | Searches | Results |

| | | |
|---|---|---|
| 1 | TS=(COVID-19 OR COVID19 OR 2019 Novel Coronavirus OR 2019-nCoV OR nCoV-19 OR Coronavirus Disease 2019 OR Coronavirus Disease-19 OR SARS Coronavirus 2 OR SARS-CoV-2 OR Severe Acute Respiratory Syndrome Coronavirus 2) | 83915 |
| 2 | TI=(Singapore) OR AK=(Singapore) OR KP=(Singapore) | 22518 |
| 3 | 1 and 2 | 236 |
| 4 | Refined 3 by: PUBLICATION YEARS: ( 2021 or 2020 ) | 225 |

**Supplementary Table S5: CINAHL**

| # | Searches | Results |
|---|---|---|
| 1 | MM "COVID-19" OR MM "Coronavirus" OR MM "Coronavirus Infections+" | 20295 |
| 2 | COVID-19 OR COVID19 OR 2019 Novel Coronavirus OR 2019-nCoV OR nCoV-19 OR Coronavirus Disease 2019 OR Coronavirus Disease-19 OR SARS Coronavirus 2 OR SARS-CoV-2 OR Severe Acute Respiratory Syndrome Coronavirus 2 | 36652 |
| 3 | MM "Singapore" | 31 |
| 4 | TI "Singapore" OR SU "Singapore" | 6273 |
| 5 | 1 OR 2 | 39329 |
| 6 | (1 OR 2) AND (3 OR 4) | 232 |
| 7 | Limit 6 to published date 2020 to 2021 | 159 |

**Supplementary Table S6: Psycinfo**

| # | Searches | Results |
|---|---|---|
| 1 | MM “Coronavirus” | 1645 |
| 2 | COVID-19 OR COVID19 OR 2019 Novel Coronavirus OR 2019-nCoV OR nCoV-19 OR Coronavirus Disease 2019 OR Coronavirus Disease-19 OR SARS Coronavirus 2 OR SARS-CoV-2 OR Severe Acute Respiratory Syndrome Coronavirus 2 | 3586 |
| 3 | MM “Southeast Asian Cultural Groups” | 637 |
| 4 | TI “Singapore” OR SU “Singapore” | 2662 |
| 5 | 1 OR 2 | 3641 |
| 6 | 3 OR 4 | 3257 |
| 7 | 5 AND 6 | 9 |
| 8 | Limit 7 to published date 2020 to 2021 | 9 |

| **Supplementary Table S7: Google Scholar** | | |
|---|---|---|
| # | Searches | Results |
| 1 | allintitle: Singapore "COVID 19" OR COVID19 OR "2019 Novel Coronavirus" OR "2019 nCoV" OR "nCoV 19" OR "Coronavirus Disease 2019" OR "Coronavirus Disease 19" OR "SARS Coronavirus 2" OR "SARS CoV 2" OR "Severe Acute Respiratory Syndrome Coronavirus 2" (date filter set as 2020-2021) | 380 |
